# Asymmetric trends in seasonal temperature variability in instrumental records from ten stations in Switzerland, Germany and the UK from 1864 to 2012

**DOI:** 10.1002/joc.4326

**Published:** 2015-04-02

**Authors:** Michael Matiu, Donna P. Ankerst, Annette Menzel

**Affiliations:** ^1^ Fachgebiet ÖkoklimatologieTechnische Universität MünchenFreisingGermany; ^2^ Lehrstuhl für Mathematische Modellierung biologischer SystemeTechnische Universität MünchenGarchingGermany; ^3^ Institute for Advanced StudyTechnische Universität MünchenGarchingGermany

**Keywords:** Climate change, quantile regression, Europe, robust measures, long‐record observations, temperature extremes, Alpine region

## Abstract

While the rise in global mean temperature over the past several decades is now widely acknowledged, the issue as to whether and to what extent temperature variability is changing continues to undergo debate. Here, variability refers to the spread of the temperature distribution. Much attention has been given to the effects that changes in mean temperature have on extremes, but these changes are accompanied by changes in variability, and it is actually the two together, in addition to all aspects of a changing climate pattern, that influence extremes. Since extremes have some of the largest impacts on society and ecology, changing temperature variability must be considered in tandem with a gradually increasing temperature mean. Previous studies of trends in temperature variability have produced conflicting results. Here we investigated ten long‐term instrumental records in Europe of minimum, mean and maximum temperatures, looking for trends in seasonal, annual and decadal measures of variability (standard deviation and various quantile ranges) as well as asymmetries in the trends of extreme versus mean temperatures via quantile regression. We found consistent and accelerating mean warming during 1864–2012. In the last 40 years (1973–2012) trends for Tmax were higher than for Tmin, reaching up to 0.8 °C per 10a in spring. On the other hand, variability trends were not as uniform: significant changes occurred in opposing directions depending on the season, as well as when comparing 1864–2012 trends to those of 1973–2012. Moreover, if variability changed, then it changed asymmetrically, that is only in the part above or below the median. Consequently, trends in the extreme high and low quantiles differed. Regional differences indicated that in winter, high‐alpine stations had increasing variability trends for Tmax especially at the upper tail compared to no changes or decreasing variability at low altitude stations. In contrast, summer variability increased at all stations studied.

## 1 Introduction

Evidence of global warming of the climate system has been unequivocal: there have been warmer and/or fewer cold days and additionally warmer and/or more frequent hot days in the recent decades [Intergovernmental Panel on Climate Change (IPCC), 2013]. In addition to mean warming, countries across the world are currently facing an increase in the frequency and intensity of temperature extremes, which is of great concern since extreme events have had and will continue to have the greatest impact on socio‐economies (Easterling *et al.*, 2000), human health (e.g. O'Neill and Ebi, 2009), and terrestrial ecosystems (e.g. Trigo *et al.*, 2006; Gloning *et al.*, 2013; Reyer *et al.*, 2013). However, temperature extremes are more sensitive to changes in variability rather than changes in mean conditions (Katz and Brown, 1992) and asymmetry, that is the skewness of the distribution, also plays a crucial role in predicting extremes (Ballester *et al.*, 2010). Previous studies implementing schematic graphs of normally distributed temperatures have illustrated how increases in mean temperature, variance, and both could affect extreme temperatures at the tails of the temperature distribution (Beniston and Goyette, 2007; Figure 2.32 in Folland *et al.*, 2001; Meehl *et al.*, 2000). However, recently, the special report on extreme events (SREX) of the IPCC updated the schematic graph to include the possibility of changes in symmetry (see Figure SPM.3 in IPCC, 2012). But the latest Assessment Report (AR5) of the IPCC, published shortly after the SREX, shows again the older symmetric depiction (Figure 1.8 in Cubasch *et al.*, 2013). Since the beginning of the ‘variability issue’ many climate‐related publications have attributed changes in the frequency and intensity of extreme events to both warming and increased variability. The recent accumulation of high‐profile extreme events, such as the European heat waves in 2003 and 2006–2007, the Australian summer of 2012–2013, the Northern Hemisphere 2010 heat wave, and the Europe 2009 and Argentina 2007 cold waves, have perpetuated this hypothesis.

The detection‐attribution algorithm (Hegerl *et al.*, 2004), previously used to estimate the anthropogenic influence on warming of mean temperatures, was extended to temperature extremes using extreme value theory. A significant human influence was found globally for warming of the warmest night, coldest days and coldest nights (Christidis *et al.*, 2005; Shiogama *et al.*, 2006) as well as for the warmest days (Christidis *et al.*, 2011). Regional extreme temperatures were also significantly influenced by humans (Zwiers *et al.*, 2010; Min *et al.*, 2013; Wen *et al.*, 2013) and land use change was found to be of particular importance for changes in warm extremes (Christidis *et al.*, 2013). By analyzing six temperature reconstructions, Rybski *et al.* (2006) found that part of the recent warming could not be attributed to natural variability.

Notwithstanding an accumulation of observed data on extreme events, the question of whether such events have been caused by shifts in mean temperature alone versus additionally by shifts in variability remains to date unresolved. There is still an ongoing debate concerning whether and how variability has changed on a global scale (Easterling *et al.*, 2000; Hansen *et al.*, 2012; Rhines and Huybers, 2013). Correct assessment of climate variability and extremes is of paramount importance for the tools and methods required for applied climate impact research, including the construction of weather generators, the downscaling of model outputs, risk assessment, and the determination of experimental settings for ecological climate change impact studies (Thompson *et al.*, 2013).

As a first step for ascertaining the role of variability in the rise of extreme temperature events, one needs to understand if and how variability has changed over the past decades. This goal is not trivial for several reasons. First, variability of observations may be described on different time scales, such as daily, monthly, or yearly and over different time spans, such as over a decade or several decades. Simple measures for variability translate to approximate measures of the spread of a probability distribution describing the theoretic behavior of the observations, such as the commonly used normal distribution. They include, but are not limited to, the sample variance (sample standard deviation), which assumes symmetric behavior around the mean, and sample quantile ranges, such as the difference between the 0.975 and 0.025 sample quantiles, which describe the interior 95% portion of the underlying probability distribution. More complicated measures of variability could be characterized by examining full probability densities that are not assumed to be normal, such as flexible mixtures of distributions for bimodal or skewed observations. Once the definition of variability has been established a second complication is that the rate of extreme events may depend in a complicated manner on changes in both the mean and the variability. For example, in their theoretical framework, Rahmstorf and Coumou (2011) concluded that the number of heat waves depended non‐linearly on the ratio of the warming trend to the short‐term standard deviation. Furthermore, the long‐term correlations also have an influence on extreme value statistics (Eichner *et al.*, 2006).

Numerous studies have analyzed changing trends in variability and arrived at different conclusions; a collection of these are listed in Table 1. Based on re‐analysis of global data and concentrating on inter‐annual fluctuations, Huntingford *et al.* (2013) found varying regional patterns but no overall change in temperature variability over the past 44 years. Similar results were found using gridded station data (Parker *et al.*, 1994; Donat and Alexander, 2012). Focusing only on the June‐July‐August (JJA) summer season, Hansen *et al.* (2012) demonstrated an increase in variability globally over the past 60 years. Scherrer *et al.* (2005) confirmed an increase in variability during the summer season in Europe, a decrease in winter and spring, and no change in fall. Based on recent historical station data, Klein Tank *et al.* (2005) showed patterns of increasing and decreasing variability of mean temperatures in Europe depending on station and season, while Simolo *et al.* (2012) found that minimum and maximum temperatures showed no change except for summer maximum temperatures in one of three regions. In the case of Australia (Collins *et al.*, 2000) overall decreases were found only for minimum temperatures, while for mean and maximum temperatures, trend signs showed regional heterogeneity. For the East Asia and south Pacific regions (Griffiths *et al.*, 2005), no overall change was found, although some stations showed significant decreases in variability of minimum and maximum temperatures. In a comprehensive station network in the United States, China, former Soviet republic and Australia, mean temperature variability did not change, although it declined for some stations (Karl *et al.*, 1995). For Argentina, Rusticucci and Barrucand (2004) found decreasing variability of minimum and maximum temperatures in summer (DJF) and increasing variability in winter (JJA), although most changes were not significant. For the Tibetan Plateau, overall inter‐annual variability of mean temperatures increased, however, some stations also showed no change or decreases (Song *et al.*, 2014). Two stations in Switzerland showed no change in variability of minimum and maximum temperatures over 104 years (Beniston and Goyette, 2007). Based on more than 100 years of station data, Della‐Marta *et al.* (2007) observed an increase in variability of maximum temperatures during the summer season in Western Europe.

**Table 1:** Overview of the recent literature findings on temperature variability. Data origin indicates whether historical observational records (stations; number thereof in brackets; multiple numbers denote different time frames and/or regional availability) versus a climate data product (gridded or re‐analysis; product name in brackets) was used. Abbreviations: CDF (cumulative density function), DJF (season: December, January and February), GEV (generalized extreme value distribution), JJA (season: June, July and August), NA (not applicable), NH/SH (Northern/Southern hemisphere), PDF (probability density function), SD (standard deviation), Qxx (0.xx quantile), Var (Variability).

Article	Data origin (number)	Time frame	Region	Var‐measure	Time base for Var‐measure	Temperature parameter	Method summary	Results
Beniston and Goyette (2007)	Station (2)	1901–2004	Switzerland	variance	year	min, max	5‐point moving average; linear regression	No change.
Collins *et al.* (2000)	Station (88)	1880–1996	Australia	SD	year	min, mean, max	daily anomalies (based on 61–90 for each day); area‐weighted average; linear regression	Overall decrease only for Tmin. Majority of stations decreased (most of them significantly); regional heterogeneity of trend sign.
Della‐Marta *et al.* (2007)	Station (54)	1880–2005	Western Europe	(F(0.9) ‐ F(0.1))/2, F is CDF of fitted GEV	14 years (only JJA)	max	standardize each station by mean and SD (1906–1990); split in nine 14‐year periods; piecewise linear detrending; fit GEV to each station and period; compute Var measures; robust linear model of measures of all stations inside a region	Significant increase.
Donat and Alexander (2012)	Gridded [HadGHCND]	1951–2010	global (land)	variance	30 years (all seasons)	min, max	split in two periods (51–80, 81–10; daily anomalies of each period; empirical PDF of each grid box/spatial aggregation; F‐test for change in variance	Spatial heterogeneity (increases and decreases). Mostly non‐significant changes.
Griffiths *et al.* (2005)	Station (89)	1961–2003	East Asia, South Pacific	SD	year	min, max	linear regression	Some significant decreases: more for Tmin than Tmax; almost no significant increases. Majority of stations no change.
Hansen *et al.* (2012)	Gridded [GISS‐GHCNv2]	1951–2010	global (land and sea)	empirical PDF	10 years (only JJA)	mean	standardize each time series by mean & sd (of 1951–1980); empirical PDF	Increase in variability
Huntingford *et al.* (2013)	Re‐analysis [ECMWF ERA‐40]	1958–2001	global (land and sea)	empirical PDF	10‐13 years	Mean	Mean yearly temp; yearly standardized anomalies (from mean & sd of each 10 yr period); empirical PDF	No global change Regional patterns with increase/decrease
Karl *et al.* (1995)	Station (187/ 223/197/40)	1911–92/ 1935–89/ 1952–89/ 1961–93	USA/China/Former Soviet Union/Australia	Mean of daily running difference per period	1, 2, 5, 10, 30 days per season per year	min, mean, max	Daily anomalies (from third‐order harmonics); running difference per period (not for yearly variability); area‐averaged to country	Mostly no change. Some significant decreases.
Klein Tank *et al.* (2005)	Station (185)	1946–99	Europe	(Q90 – Q10)/2	season & year	mean	Running percentiles (5 day window); average over season/year; *t*‐test for two periods (before and after 1975)	Significant increases and decreases depending on station and season.
Parker *et al.* (1994)	Gridded [MOHSST5 & CRU data]	1954–93	global (land and sea)	Variance	20 years (for each season)	mean	Variance of period for each grid and season	No overall change
Reich (2012)	Station (188/343)	1931–2009/ 1980–2009	south‐east US	NA	NA	min, mean, max	Hierarchical Bayesian approach; spatiotemporal and simultaneous quantile regression	Locations with increasing and decreasing variability, more spatial variability for Tmin and Tmean, almost none for Tmax.
Rusticucci and Barrucand (2004)	Station (not mentioned)	1959–98	Argentina	SD	Season (JJA and DJF)	min, max	Linear regression	Tmin in summer (DJF) decreasing, partly also Tmax. Tmax in winter (JJA) increasing, partly also Tmin. Mostly non‐significant changes.
Scherrer *et al.* (2005)	Gridded [CRUTEM2v]	1961–2004	Europe [all land grid 3°W‐27°E and 44‐55°N]	SD	30 years (each season)	mean	Piecewise detrending; 30 year running SD; standardize by SD of 1961–90; mean vs SD change by time (bootstrapped confidence)	Significant increase in summer, decrease in winter and spring, no change in fall
Simolo *et al.* (2012)	Station (69)	1961–2007	Europe	Second L‐moment	Year, DJF & JJA	min, max	Standardize each series by mean of 1961–90; average into 3 regional series; compute L‐moments for every season/year; linear regression	No change except for JJA Tmax in one region
Song *et al.* (2014)	Station (63)	1960–2008	Tibetan Plateau	SD	Year/10 years	mean	Intra‐annual: linear regression of SD; inter‐annual: linear regression of annual means, SD of running 10 year residuals; regional series: area‐weighted mean	Significant decrease/increase in intra‐annual/inter‐annual variability for the whole region. For individual stations regional patterns with increases, no change and decreases.

It is difficult to compare the results of these studies due to the different temperature measures and datasets used. Therefore, using high‐quality homogenized temperature records of ten stations in Europe dating back 150 years, this report investigates the effects of different time bases for the variability measures, time frames to detect trends, statistical models and measures of variability for three variables (daily minimum, mean, and maximum temperature) on qualitative and quantitative inferences concerning changes in variability.

## 2 Data and methods

### 2.1 Data

**Figure 1:**
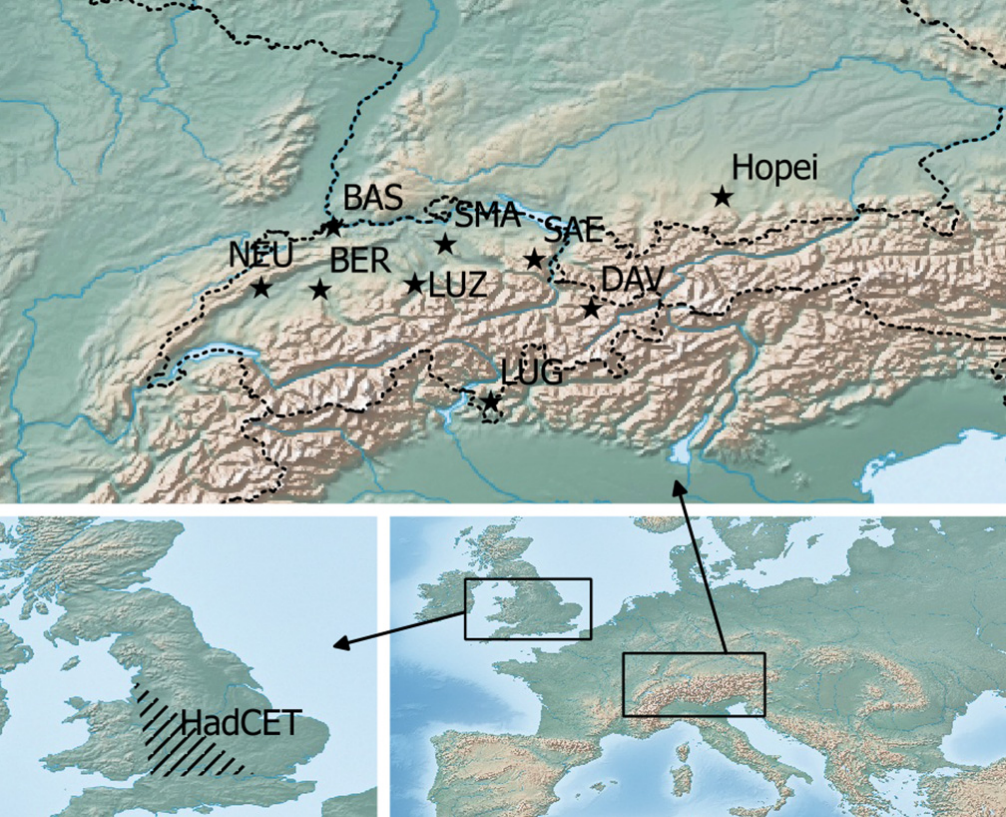
Map of available stations. Top panel shows the eight Swiss stations and Hohenpeissenberg (Hopei), bottom left is a rough representation of the area covered by the Hadley Centre Central England Temperature (HadCET) series. Station names are provided in Table 2.

Minimum, mean, and maximum daily temperature data were available for eight Swiss stations with long‐term records (115–149 years) from the SwissMETEO website (Begert *et al.*, 2005). In addition, data from the Hadley Centre Central England Temperature (HadCET) composite time series (Parker *et al.*, 1992) and the oldest mountain climate station in southern Germany, Hohenpeissenberg (DWD, German Meteorological Service), were available for analysis with 135 and 131 years of data, respectively. The HadCET series is a composite of multiple stations in central England. The region spans a roughly triangular area between London, Bristol and Lancashire; for a complete list of stations see Parker *et al.* (1992). The ten stations were roughly grouped according to altitude and whether they lay North or South to the Alps‐ridge into three categories: High‐Alps, northern Low‐Alps and Rest (Lugano in the Southern Alps and Central England). Table 2 provides summary data for the individual stations and Figure 1, a map. The collective dataset comprised daily temperature readings starting with 1864 and ending with 2012.

**Table 2:** Summary of station details. Station name is followed by country abbreviation in parentheses (CH = Switzerland, DE = Germany, GB = United Kingdom). The last column shows the number of years of available data for minimum/mean/maximum temperatures. Geographic coordinates of HadCET are an indicator of the series' regional cover. Group abbrevations: High‐Alps (High), northern Low‐Alps (Low) and Rest comprises Lugano in the southern Alps as well as Central England.

Station ID	Station name	Longitude	Latitude	Altitude [m a.s.l.]	Group	Years of data [min / mean / max]
BAS	Basel / Binningen (CH)	7°35'E	47°32'N	316	Low	115/149/115
BER	Bern / Zollikofen (CH)	7°28'E	46°59'N	552	Low	149/149/149
DAV	Davos (CH)	9°51'E	46°49'N	1594	High	123/137/123
HadCET	Central England (GB)	0°‐3°W^a^	51°–54°N^a^	0‐200^a^	Rest	135/135/135
Hopei	Hohenpeissenberg (DE)	11°01'E	47°48'N	1000	High	131/131/131
LUG	Lugano (CH)	8°58'E	46°00'N	273	Rest	148/149/148
LUZ	Luzern (CH)	8°18'E	47°02'N	454	Low	127/132/127
NEU	Neuchâtel (CH)	6°57'E	47°00'N	485	Low	148/149/148
SAE	Säntis (CH)	9°21'E	47°15'N	2502	High	121/129/112
SMA	Zürich / Fluntern (CH)	8°34'E	47°23'N	555	Low	131/149/131

a Approximate.

Except for minimum temperatures at the Swiss stations, all temperature series were homogenized in order to reduce non‐meteorological effects, such as changes in site location, measurement devices, measurement times etc.; the processes are described in detail in the original and subsequent reports (Parker *et al.*, 1992; Begert *et al.*, 2005; Parker and Horton, 2005). No statistical outliers were found and no further data standardization was performed due to the high quality standards of the data. A small amount of the daily data was missing (<0.5% per station and temperature variable, i.e. minimum, mean or maximum temperature), which was filled by single imputation (Rubin, 1978; Baraldi and Enders, 2010). Single rather than multiple imputation was used following the recommendation that the number of necessary imputations be set as the percent of missing information, which in this case was less than 1% (Bodner, 2008; White *et al.*, 2011). To perform the imputation of missing values, linear models were fit with the missing temperature variable as outcome, e.g. maximum temperature, and the other temperature variables as predictors, e.g. mean and minimum temperature, within 10 years with no missing outcomes or predictors. Rather than using the mean from the regression, the missing value was replaced with a randomly sampled value from a normal distribution with predicted mean and variance from the imputation regression, corresponding to imputation from the predictive distribution (Brick and Kalton, 1996).

Analyses were done on seasonal, annual and decadal scale. The daily observations were grouped into meteorological seasons: December‐January‐February (DJF, winter), March‐April‐May (MAM, spring), June‐July‐August (JJA, summer) and September‐October‐November (SON, fall). Incomplete winter seasons (at the start and end of the series) were removed. The most recent decade was 2003–2012 preceded by 10‐year periods (1993–2002, and so forth). An incomplete decade at the beginning of the series was removed from the decadal analysis. Increasing temperature within a decade might inflate the decadal variability measures. We also calculated the decadal variability measures after de‐trending each year with its annual mean, but found results to be identical to the original measures, and thus used the raw values for the analysis.

### 2.2 Methods

In the following, measures for temperature variability are defined. We first motivate the distinction between seasonal and annual measures of variability. Then robust quantile‐based variability measures are compared to the symmetric standard deviation. Afterwards we describe the structure of the linear mixed effects model used to detect changes in variability over time. In order to study how changes in variability and mean relate to changes in extremes, we use quantile regression, as described further below.

#### 2.2.1 Seasonal vs. annual variability

**Figure 2:**
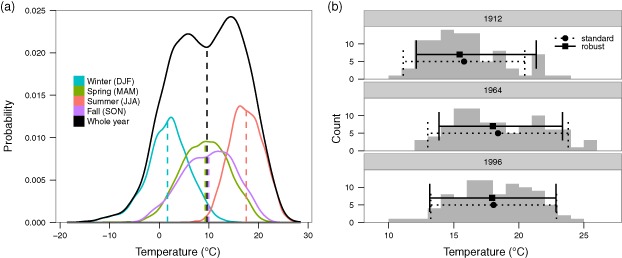
(a) Densities of daily mean temperatures from 1961 to 1990 at the Basel station according to season and for the whole year. Vertical dashed lines denote the mean of the distribution. (b) Histograms of daily mean temperatures in summer (JJA) for the Basel station for 3 distinct years. Superimposed are range bars: Standard implies the mean as center point and 1.64 times the standard deviation as a 90% confidence interval as would be estimated assuming a normal distribution. Robust has the median as its center point and the span between the 0.05th and the 0.95th quantiles as the interval limits (i.e. empirical 90%).

Changes in seasonal variability of temperatures may not correspond to similar changes in annual variability. Consequently, it is critical to compare the same type (seasonal versus annual) when discussing results in the context of other studies. To illustrate, Figure 2(a) shows the daily mean temperature distribution of Basel from 1961 to 1990. The mean of the annual temperature distribution is approximately equal to the average over the four means of the seasonal temperature distributions. This fact is supported by principles in statistics, that the grand mean (in this case over the annual temperatures) is equal to the mean of the sub‐means (in this case over the component seasons), if the sub‐means are of equal size. However, the same general principle does not hold statistically for the variance by the law of total variance, also known as Eve's law. Specifically, the variance of temperatures on the annual scale depends on both the seasonal variances as well as the seasonal means. To see this, picture in Figure 2(a) shifting either the individual season means or increasing or decreasing the individual season variances: both will affect the overall spread of the annual distribution in any multitude of ways. Thus we used seasonal as well as annual measures of variability in all our analyses.

#### 2.2.2 Normal‐based vs. asymmetric and robust approaches to variability

In addition to or in lieu of the commonly used standard deviation (SD), there are more robust measures to quantify variability that can also handle asymmetrical distributions. These do not assume a symmetric normal distribution where variability for temperatures above the mean is assumed to be the same as that for temperatures below the mean, so that a single measure, the SD, applies to both. Rather, they rely on sample quantiles of the observed temperatures, say the difference between the 2.5th and 97.5th largest temperatures in the observed dataset (all observed temperatures are ordered from smallest to largest to make this ranking). We term these approaches as robust because they yield the same results as SD‐based symmetric confidence intervals (CIs), such as 95% CIs (mean ± 2 × SD), when the distribution of temperatures does indeed follow a normal distribution, but also yield accurate results when the true distribution of temperatures does not follow a normal distribution, and in fact under whatever the true underlying distribution of temperatures happens to be (see Stigler (2010) for a history of robust statistics). These approaches are also robust to outliers, which are individual temperature values that are extremely high or low. The 0.025 quantile of the dataset (that temperature below which 2.5% of the observations fall below it) is not affected whether the lowest temperature in the dataset was its observed value or an extreme event with 10° below it. Figure 2(b) shows specific examples of how robust asymmetric interval ranges can differ from symmetric CIs. For our analyses we used both, the SD as well as robust and potentially asymmetric quantile ranges.

#### 2.2.3 Modeling changes in mean temperature and variability over time

Climate change affects all aspects of the temperature distribution, from central mean or median aspects, to variability, to extremes. In this section we study trends in mean, median, and variability measures, in the next, we look at associations between these changes. We begin with mean trends, using as response variables the seasonal, annual and decadal means calculated from daily minimum, mean and maximum temperature values. We used data from all stations together in one mixed‐effects model:

$$\begin{array}{cc} Y_{st,t} & =\left(\beta_{0}+b_{0,st}\right)+\left(\beta_{1}+b_{1,st}\right)*t+\epsilon_{st,t}\;,\\ & b_{0,st}\;∼\;N\left(0,,,\sigma_{0}^{2}\right),\;b_{1,st}\;∼\;N\left(0,,,\sigma_{1}^{2}\right),\\ & \epsilon_{st,t}\;∼\;N\left(0,,,\sigma_{\epsilon }^{2}\right)\text{,} \end{array}$$

where Y_st,t_ is the seasonal/annual/decadal mean at station st at time t, β_0_ is the common intercept of all stations, b_0,st_ is the deviation of each station from the common intercept (treated as a random variable in the model), β_1_ is the common time trend in Y for all stations, b_1,st_ is the deviation of each station from the common time trend (treated as a random variable in the model), t is the time of Y (for seasonal and annual means it is the year centered at the median year 1945 and divided by ten; for decadal means it is the decade; thus time trends are per decade in all three cases) and ϵ_st,t_ is the independent normally distributed within‐station error. The deviations of each station from the common intercept and trend (b_0,st_ and b_1,st_, respectively) account for differences among the stations in terms of both the mean and trend with time in Y. We additionally fit the model restricted to the last 80 and 40 years of available data, as the last third of the data (1972–2012) corresponded to a period of strong temperature increase that occurred since the mid‐1970s, and the last 80 years (1933–2012) additionally included a previous period of slight cooling. Residual plots were inspected to verify model assumptions. The residuals had significant autocorrelation up to order 3, thus an autoregressive process of order 3 was added to the model residuals, i.e. ϵ_st,t_ = ϕ_1_ ϵ_st,t − 1_ + ϕ_2_ ϵ_st,t − 2_ + ϕ_3_ ϵ_st,t − 3_ + u_st,t_ with estimated autoregressive coefficients ϕ_1_, ϕ_2_, ϕ_3_ and independent within‐station errors $u_{st,t}\;∼\;N\left(0,,,\sigma_{\epsilon }^{2}\right)$.

A more robust estimate of mean conditions is the median, thus we repeated the above process with seasonal, annual and decadal medians of daily temperatures (replace Y in the model above with the median instead of the mean).

The same model structure was used to detect changes in variability. We took the standard deviation (SD) and three different quantile ranges to account for different measures of the variability of the temperature distribution: the central 50% region (difference between the 0.75 and 0.25 quantile, Q75‐25), the central 90% region (0.95–0.05 quantile, Q95‐05) and the central 95% region (0.975–0.025 quantile, Q975‐025). In order to detect possible asymmetries related to changes in the spread or variability of the probability distribution, we further divided the central 90% region into a lower 45% region (0.05–0.50 quantile, Q50‐05) measuring the spread of the lowest temperatures and an upper 45% measure (0.50–0.95 quantile, Q95‐50) measuring the spread of the highest temperatures to better identify how variability changed, e.g. by a symmetric increase in variability for low and high temperatures, for only high temperatures, and so forth. With all measures of variability, the model residuals were not autocorrelated, thus the models were fit without the autoregressive error structure. Table 3 provides a summary of all measures used as model outcome variables.

**Table 3:** Summary of measures used to detect changes in mean conditions and variability over time. Each of these was calculated out of daily minimum, mean and maximum temperatures on a seasonal, annual and decadal basis. They served as response variable (Y) in the mixed‐effects model. Last column shows whether the model residuals had significant autocorrelation and if so, up to what lag.

Measure	Description	Autocorrelation
Mean	sample mean	Yes, up to lag 3
Median	sample median (i.e. the 0.50 quantile)	Yes, up to lag 3
SD	sample standard deviation; for a normal‐distributed sample, the interval of ±1 SD around the mean holds approximately 68% of observations	No
Q75‐25	difference between the 0.75 and 0.25 quantile; length of the interval that contains the central 50% of observations	No
Q95‐05	difference between the 0.95 and 0.05 quantile; length of the interval that contains the central 90% of observations	No
Q975‐025	difference between the 0.975 and 0.025 quantile; length of the interval that contains the central 95% of observations	No
Q50‐05	difference between the 0.50 and 0.05 quantile; length of the interval that contains 45% of observations below the median, without the lowest 5%	No
Q95‐50	difference between the 0.95 and 0.50 quantile; length of the interval that contains 45% of observations above the median, without the highest 5%	No

Qualitative statements on changes in extremes could be derived by examining trends in mean temperature and variability together. If, for example, mean temperature increased, then there would be more warm and less cold extremes. If additionally variability increased symmetrically, there would be even more warm extremes, as well as more cold extremes than with merely an increase in mean temperature. This approach has multiple drawbacks. First, only qualitative and not quantitative statements are possible. Secondly, relating trends in mean to variability results in many possibilities, especially if variability changes asymmetrically, such as only in the warmer part. Thus we propose an alternative method: quantile regression.

#### 2.2.4 Joint assessment of changes in mean temperature, variability and extremes via quantile regression

Changes in the distribution of minimum, mean and maximum temperatures were detected by simultaneously examining trends in multiple quantiles thereof. Assuming that the temperature distribution is characterized by the set of 19 equally spaced quantiles (0.05, 0.10, …, 0.95), time trends of these quantiles could identify changes in distribution, for example, if the higher quantiles increased and the lower quantiles decreased, then the spread of the distribution has increased and thus also variability has increased. Also changes in extremes (i.e. the extreme quantiles 0.05 and 0.95) could be related to changes in mean temperature (i.e. the median; 0.50 quantile).

We used quantile regression (Koenker and Bassett, 1978; Koenker, 2005) to estimate linear time trends of the seasonal and annual distribution of minimum, mean and maximum temperatures for each station. To estimate time trends, we used the total amount of data which was available at each station (see Table 2) and in a second step only the last 40 years (1973–2012), in order to distinguish between long‐ and short‐term trends. Trends were estimated simultaneously for 19 quantiles (0.05, 0.10, …, 0.95) with the algorithm specified in Bondell et al. (2010) in order to ensure non‐crossing of the quantile trend lines. Crossing quantile trend lines would contradict the definition of quantiles, e.g. if the 0.95 quantile trend line crossed the 0.90 quantile trend line at a certain time, then for some years the estimated temperature at the 0.95 quantile would be below the temperature at the 0.90 quantile, which is impossible via definition.

All statistical analyses were performed in R version 3.1.0 (RCoreTeam, 2008). The quantreg‐package (Koenker, 2008) was used for quantile regression and the nlme‐package (Pinheiro et al., 2013) for linear mixed effects models. Statistical significance was assumed at the 0.05 level unless otherwise stated.

## 3 Results

### 3.1 Overall trends in mean temperature and variability measures

**Figure 3:**
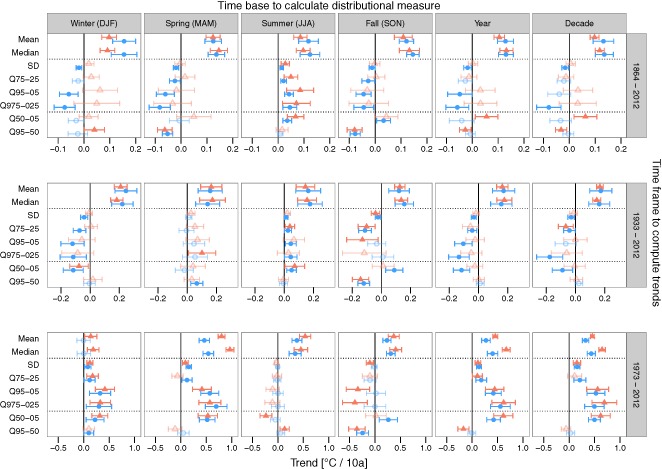
Estimated common time trend coefficients for linear mixed effects models of various distributional measures of maximum temperatures (Tmax; red triangles) and minimum temperatures (Tmin; blue circles) versus time of all stations, depending on the time base used to compute the measures (columns) and the time frame for trend estimation (rows). Error bars show 95% confidence intervals. Trends in solid lines are significant at the 0.05 level, while the transparent ones are not, i.e. zero is within the confidence bounds. SD = Standard deviation, quantile‐based measures start with Q, followed by the bounds (e.g. Q95‐05 is the range between the 0.95 and the 0.05 quantile).

Time trends of mean temperatures, median temperatures and all the measures of variability, such as SD and robust intervals (Q75‐25, Q95‐05, Q975‐025, Q50‐05 and Q95‐50) across all stations included in the study are shown in Figure 3 for Tmax and Tmin, the temperature variables of interest, and Supplementary Figures S1 for Tmean. We first present mean and median trends followed by trends in variability. We conclude with a few remarks on the individual station level.

#### 3.1.1 Mean/Median trends

Seasonal, annual and decadal mean and median trends of daily Tmax were all significantly positive. For the period 1864–2012 they were between 0.09 and 0.15 °C per 10a (all *p* < 0.001) and for the period 1933–2012 they were between 0.13 and 0.21 °C per 10a (all *p* < 0.001). For the last 40 years (1973–2012) warming was not as uniform over seasonal, annual and decadal measures as compared to the longer periods, however still significantly positive. Trends of annual and decadal means were 0.46 °C per 10a (0.41, 0.50; 95% confidence interval), while trends of annual and decadal medians were higher with 0.67 °C per 10a (0.59, 0.75) and 0.65 °C per 10a (0.58, 0.71), respectively. Seasonal trends differed even more, as for instance mean winter Tmax rose at only 0.14 °C per 10a (0.02, 0.26) compared to mean spring Tmax, which rose at 0.80 °C per 10a (0.73, 0.87). In summary, mean and median trends show consistent and accelerating warming of maximum temperatures from 1864 to 2012 and seasonally diverging trends in the period 1973–2012. Tmin trends exhibited the same patterns in general, however, during 1864–2012 winter and summer Tmin warmed more than Tmax, e.g. the mean winter Tmin trend was 0.16 (0.11, 0.20) compared to 0.10 °C per 10a (0.07, 0.13) for Tmax. In the 1973–2012 period the opposite was true, i.e. Tmin warmed less than Tmax in all seasons, as well as on annual and decadal scale. This effect was most apparent in spring, where the mean Tmin trend [0.46 °C per 10a (0.36, 0.56)] was approximately half of the Tmax trend [0.80 °C per 10a (0.73, 0.87)], and in winter, where mean Tmin did not change at all (*p* = 0.95) compared to small increases in Tmax (0.14 °C per 10a, *p* = 0.02).

#### 3.1.2 Trends in measures of temperature variability

Contrary to mean and median, trends in temperature variability did not point in the same direction for each time base, time frame and temperature variable (Figure 3). For instance, during the period 1864–2012, variability of Tmax did not change for winter, spring, fall, annual and decadal measures (*p* > 0.05), only in summer all variability measures showed a significant increase in variability (all *p* < 0.01). For the period 1973–2012, however, the opposite is true: summer variability did not change (all measures *p* > 0.05), but winter, spring, annual and decadal variability increased and fall variability decreased (all measures except Q75‐25; *p* < 0.01). While there were high differences in trends between the four measures of variability for each time base, e.g. annual SD increased at 0.12 °C per 10a (0.06, 0.17) and annual Q95‐05 increased at 0.45 °C per 10a (0.26, 0.63), the trends of each of the four measures were of similar magnitude between time bases, i.e. the seasons, year and decade. For instance, SD increased at 0.12 °C per 10a (0.06, 0.18) in winter, 0.09 °C per 10a (0.03, 0.14) in spring and 0.15 °C per 10a (0.08, 0.22) when calculated on a decadal base; Q95‐05 increased at 0.42 °C per 10a (0.23, 0.60) in winter and spring, and 0.56 °C per 10a (0.34, 0.78) when calculated by decade. In summary, there were opposing variability trends for maximum temperatures of the 1864–2012 period compared to those of the recent 1973–2012 period, and seasonal divergence of variability trends in the recent period. Variability trends for Tmin were close to Tmax during the last 40 years (1973–2012), but not so for the 1864–2012 period, where variability of Tmin decreased in winter, spring, fall and on the annual time base (all measures except Q75‐25, *p* < 0.05) compared to no changes in Tmax variability.

#### 3.1.3 Asymmetric changes in temperature variability

The increase in Tmax summer variability during 1864–2012 was accompanied by an increase in variability of the colder part of temperatures but not in the warmer part, as Q50‐05 increased and Q95‐50 did not change (*p* < 0.001 and *p* = 0.19). Annual variability during the same period did not change, however, Q50‐05 increased at 0.06 °C per 10a (0.01, 0.10) and Q95‐50 decreased to −0.03 °C per 10a (−0.05, 0.00), i.e. changes in the variability of the colder and warmer part of Tmax canceled each other out in terms of total variability. This further implies that the distribution of annual temperatures did not become wider as such, but changed shape. Even more asymmetric changes were found for Tmax during the period 1973–2012, where either only one of the two measures (Q50‐05 and Q95‐50) changed, or both changed but in opposite directions. For instance winter and spring Q95‐50 of Tmax showed no change (both *p* > 0.05), only Q50‐05 increased at 0.32 (0.16, 0.47) and 0.53 °C per 10a (0.34, 0.72), respectively. In other words, the increased variability [Q95‐05 trend of 0.42 °C per 10a (0.23, 0.60)] is only because variability of the colder part of temperatures increased, but not in the warmer part. Based on annual measures of Tmax, variability of the colder part increased much stronger [0.62 °C per 10a (0.43, 0.80)] than variability of the hotter part decreased [−0.17 °C per 10a (−0.28, −0.06)]. This yielded a smaller net increase in total variability Q95‐05 [0.45 °C per 10a (0.26, 0.63)], which masked the asymmetric changes.

Q50‐05 and Q95‐50 trends of Tmin were different than corresponding trends of Tmax in few cases, most notably during the last 40 years (1973–2012). Variability of winter Tmin during 1864–2012 and 1973–2012 changed symmetrically, as variability of the upper and lower part did not change for the 1864–2012 period (both *p* > 0.05) and increased simultaneously during 1973–2012 (both *p* < 0.05). Additionally, summer Tmin variability as well as asymmetry did not change (all *p* > 0.1), but fall showed the strongest sign of asymmetry, as Q50‐05 increased at 0.26 °C per 10a (0.08, 0.44), while Q95‐50 decreased to −0.24 °C per 10a (−0.36, −0.13), which lead to no change in the total variability (all four measures had *p* > 0.1). In summary, variability of the upper and lower part of temperatures rarely changed in the same direction, i.e. changes in total variability were driven by changes in either variability of colder or warmer temperatures and in few cases no change in total variability was caused by opposing effects of the upper and lower variability, which canceled each other out.

#### 3.1.4 Individual stations

**Figure 4:**
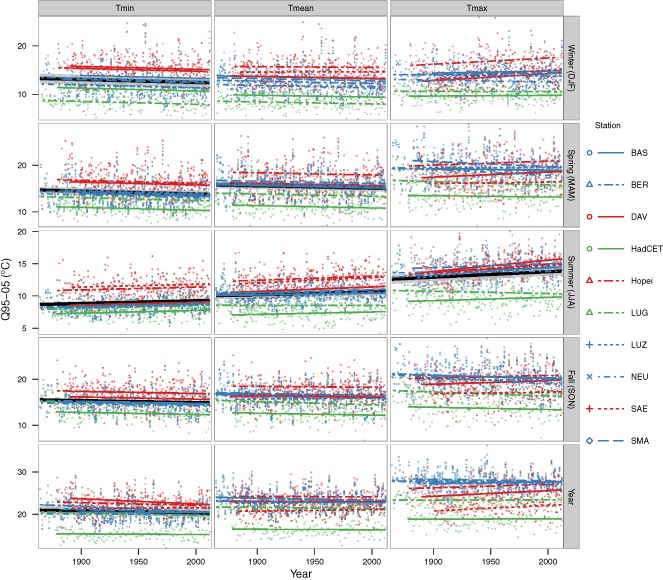
Modelled trends and raw values of Q95‐05 (difference between 0.95 and 0.05 quantile) for each station according to time base and temperature variable for all years of data. Stations were grouped into High‐Alps (red), northern Low‐Alps (blue) and South‐Alps/England (green). Overall trends for all stations combined are shown in black (95% confidence bands grey), if they are significant (see also values for Q95‐05 in Figure 3 and S1).

Individual station trajectories in the Q95‐05 measure of variability across all years of data and all three temperature variables are shown in Figure 4. For Tmin, variability trends of the individual stations were almost identical to the overall trend, however, baseline Q95‐05 was different: high alpine stations had the highest and Central England (HadCET) had the lowest baseline rate of Q95‐05 due to the maritime influence, for instance, annual variability (Q95‐05) in 1945 was 23.1 °C in Davos (DAV) and 15.3 °C in Central England (HadCET). The same applied to winter, spring and summer Tmean trends. Fall and yearly Tmean and all of Tmax trends revealed more differences among the individual stations as well as a greater magnitude of variation from the overall trend. For winter, spring and fall Tmax, individual stations showed opposing behavior: in High‐Alps stations variability increased, while in the other stations it decreased, e.g. spring Q95‐05 trend was 0.08 °C per 10a at Hohenpeissenberg (Hopei) and −0.07 °C per 10a at Lugano (LUG).

### 3.2 Simultaneous changes in temperature mean and variability and their effect on extremes

**Figure 5:**
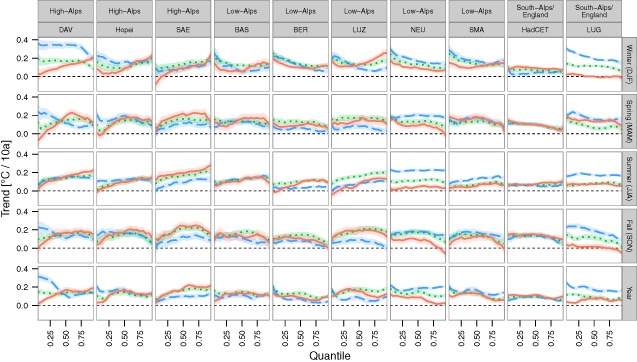
Slope‐quantile plots from quantile regression of temperature versus time for the ten stations and different seasons, as well as for the whole year as time base. Time trends were estimated for the 0.05, 0.10, … , 0.95 quantiles. Colors code the different temperature variables (Tmin = blue dashed, Tmean = green dotted, Tmax = red solid). Trends at the specific quantiles are significant at the 0.05 level, if the 95% confidence bands do not cross the dashed zero line, and not significant if they cross it. See Supplementary Figure S2 for an explanation of slope‐quantile plots.

We used quantile regression to model changes in the full temperature distribution of each station, characterized by time trends of the 0.05, 0.10, …, 0.95 quantiles of Tmin, Tmean and Tmax, in contrast to the previous section, which dealt with overall trends in ranges of quantiles. Seasonal and annual quantile trends are summarized in terms of slope‐quantile plots for each of the ten stations (Figure 5).

The stations at high elevation (DAV, SAE and Hopei) exhibited an increasing variability of the Tmax distribution during 1864–2012, as lower quantiles had smaller trends than high quantiles, i.e. coldest Tmax warmed less than hottest Tmax. For instance coldest summer Tmax, i.e. the 0.05 quantile, at Saentis (SAE) increased at 0.11 °C per 10a (0.07, 0.15), while the hottest temperatures, i.e. the 0.95 quantile, increased at 0.28 °C per 10a (0.24, 0.33). Actually, coldest Tmax did not change for the high‐Alpine stations in some seasons and annually, where e.g. 0.05 quantile trends were not significantly different from zero (p > 0.05). Thus the increase in variability totally offset the effect of mean warming for coldest Tmax at these high elevation sites. This effect also occurred for Tmin at the highest site (SAE) in all seasons except winter, i.e. coldest Tmin did not warm in spring, summer and fall (all p > 0.05).

Sites at lower elevation (BAS, BER, LUZ and NEU) showed an increase in variability mainly for Tmax in summer, which was due to smaller warming trends of the lowest quantiles compared to the median. Higher quantiles had trends similar to the median, thus for these stations the increase in hot Tmax extremes is in accordance to median (or mean) trends. For instance with summer Tmax at BER, the trend of the 0.05 quantile was −0.01 °C per 10a (−0.04, 0.02), which is lower than the median trend of 0.09 °C per 10a (0.07, 0.12), which itself is almost identical to the trend of the 0.95 quantile 0.09 °C per 10a (0.06, 0.12).

For LUG, which is located south of the Alps ridge, trends for Tmin were higher than for Tmax for all quantiles and seasons. Additionally, winter, spring and fall Tmin showed reduced variability, as warming of the coldest temperatures was higher than for hottest temperatures, for example in winter the 0.05 quantile of Tmin increased at 0.31 °C per 10a (0.27, 0.34), while the 0.95 quantile increased less at only 0.14 °C per 10a (0.12, 0.17).

**Figure 6:**
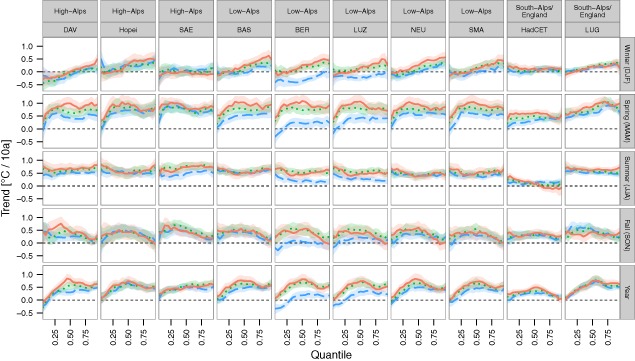
Same as Figure 5, but only for the last 40 years of available data (1973–2012).

Restricting the analyses to the last 40 years (Figure 6) yielded trends that differed more between quantiles and seasons, thus implying asymmetric changes in the temperature distribution. For instance, in spring lowest temperatures warmed much less than median and higher temperatures, and especially for Tmin, most stations (except BER and LUG) showed no change at all in lowest temperatures (0.05 quantile trends: all p > 0.05). Summer trends showed significant warming in all parts of Tmin and Tmax (trends for all quantiles positive, all p < 0.05) except for HadCET. Still asymmetries occurred especially for Tmax, as median trends were in most cases lower than trends at the coldest and highest quantiles, e.g. at SMA the median trend was 0.50 °C per 10a (0.31, 0.69) and trends for the 0.05 and 0.95 quantile were 0.68 (0.44, 0.93) and 0.67 °C per 10a (0.42, 0.9), respectively. Fall trends showed the opposite, i.e. median trends were higher than trends at the lowest and highest quantiles, as e.g. Tmax at NEU had a median trend of 0.60 °C per 10a (0.34, 0.86) compared to 0.30 (0.00, 0.60) and 0.03 °C per 10a (−0.22, 0.28) trends of the 0.05 and 0.95 quantile. In summary, the temperature trends were not the same for the colder and warmer parts of seasonal temperatures, and especially in the last 40 years, extreme temperatures did not change according to median trends.

## 4 Discussion

Conclusions from this analysis of roughly 150 years of data from ten different stations in Europe were that variability of temperature did change over this period, but not equally in all seasons and that the change in temperature variability was not symmetric in most cases. The asymmetry makes it harder to relate mean temperature trends to variability trends in their compound effect on changes in extreme temperatures. Looking directly at trends in multiple temperature quantiles, changes in mean, variability and extremes and their interdependencies were simultaneously compared.

We used multiple measures of variability and found that time trends for the different measures followed the same direction, however, their magnitude varied (Figure 3). The standard deviation (SD) had the smallest absolute trends out of the four measures and hence the shortest confidence bands, followed by the robust 50% quantile range (Q75‐25) with slightly larger trends and confidence intervals. The 90% (Q95‐05) and 95% (Q975‐025) quantile ranges had the largest absolute trends and confidence intervals. The differences in magnitude and confidence bands of trends tracked the size of the variability measure: SD was the smallest variability measure due to the normality approximation, followed by the quantile ranges, whose length increased with the quantile size. After standardizing the outcome (to zero mean and unit standard deviation), there was no longer a difference between the magnitude of trends (see Supplementary Figure S3).

But the notion of temperature variability has shortcomings since it is a compound measure that combines both the effects in the lower and upper parts of the temperature distribution. Our comprehensive analysis showed that changes in temperature variability can be more precisely characterized by isolating changes in the upper and lower ends of the temperature distribution, i.e. by changes in the coldest temperatures versus changes in the warmest. The observation of no trend in variability could be the result changes in opposite directions at the upper and lower ends that have canceled each other out. The most commonly used measure of SD based on the assumption of an underlying symmetric normal distribution failed in this regard, as e.g. during 1973–2012 summer SD of Tmax showed no change (*p* = 0.14), while the variability of the lower part (Q50‐05) decreased and variability of the upper part (Q95‐50) increased (both *p* < 0.01; see also Figure 3).

By separating variability into an upper and lower part, we found that asymmetries occurred on different time scales. First, trends in mean and variability were not uniform across seasons (Figure 3), thus the annual temperature distribution did not have symmetric changes in variability, as the upper and lower variability measures had opposing trends, e.g. during 1864–2012, where variability of the upper part of annual temperatures increased and variability of the lower part decreased (both *p* < 0.05). This is in accordance to previous studies, which found that changes differed by season (see e.g. Caesar *et al.*, 2006 and Scherrer *et al.*, 2005). On the other hand, using an annual or decadal time base to compute variability measures made almost no difference in the estimated trends (Figure 3). Secondly, seasonal variability of the upper and lower parts of the temperature distribution did not change simultaneously and in the same direction (Figure 3), thus changing the shape of the seasonal distribution of minimum and maximum temperatures. Third, Tmax and Tmin had different time trends in mean and variability, especially during the 1973–2012 period, implying that warm and cold temperatures changed at different rates. Similar conclusions were reported by Alexander *et al.* (2006) and Brown *et al.* (2008), who analyzed a global dataset of extreme temperature indices and found higher warming rates for minimum than for maximum temperatures. However, trends of minimum temperatures reported in our study should be taken with caution, especially for the longer periods, as the minimum temperature series from 8 of 10 studied stations were not homogenized. In sum, we draw a similar conclusion to that reported in Seneviratne *et al.* (2012), namely that in addition to mean and variability, multiple aspects of the temperature distribution play an important and regionally varying role toward predicting extreme events in climate.

An appealing alternative to separately reporting temperature changes in terms of mean and variability is the simultaneous quantile regression analysis, which do not assume a symmetric Normal distribution for temperature, but rather analyze trends at multiple quantiles ranging from the lowest temperatures to the highest (Figures 5 and 6). Thus median trends, asymmetric changes in variability and trends in extreme quantiles could be compared directly. This approach could be extended in the future in order to analyze overall trends instead of station‐specific trends. Extending the study area to all of Europe and other continents would provide further insight whether asymmetric changes in variability are a special feature of the Alpine region in Europe, or a more general phenomenon.

## Supporting information


**Figure S1.** Estimated common time trend coefficients for linear mixed effects models of various distributional measures of mean temperature versus time of all stations, depending on the time base used to compute the measures (columns) and the time frame for trend estimation (rows). Error bars show 95% confidence intervals. Trends in solid lines are significant at the 0.05 level, while the transparent ones are not, i.e. zero is within the confidence bounds. SD = Standard deviation, quantile‐based measures start with Q, followed by the bounds (e.g. Q95‐05 is the range between the 0.95 and the 0.05 quantile).

**Figure S2.** Quantile Regression example of slope‐quantile plots.

**Figure S3.** Estimated common time trend coefficients for linear mixed effects models of various distributional measures of minimum and maximum temperature versus time of all stations, depending on the time base used to compute the measures (columns) and the time frame for trend estimation (rows). Error bars show 95% confidence intervals. Trends in solid lines are significant at the 0.05 level, while the transparent ones are not, i.e. zero is within the confidence bounds. SD = Standard deviation, quantile‐based measures start with Q, followed by the bounds (e.g. Q95‐05 is the range between the 0.95 and the 0.05 quantile), but responses scaled to zero mean and unit standard deviation.
