# Supplementary figures and images for: Asymmetric trends in seasonal temperature variability in instrumental records from ten stations in Switzerland, Germany and the UK from 1864 to 2012
Source: Int J Climatol. 2015 Apr 2;36(1):13–27. doi: 10.1002/joc.4326 (PMC4950111; doi:10.1002/joc.4326)

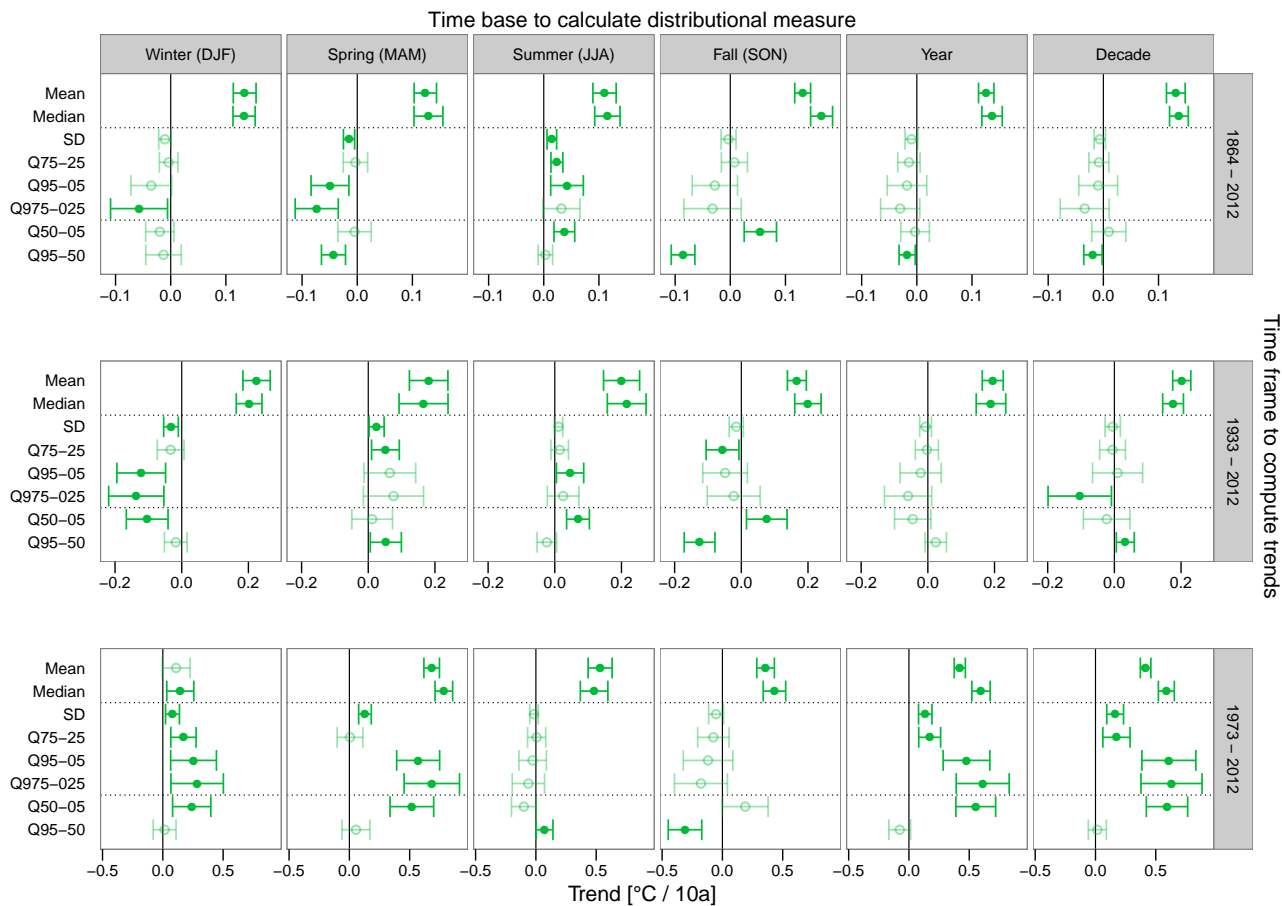

Figure S1: Same as Figure 3, but for mean temperatures ( $T_{\text{mean}}$ ).

Supplement: Supplementary file 1 — Figure S1. Estimated common time trend coefficients for linear mixed effects models of various distributional measures of mean temperature versus time of all stations, depending on the time base used to compute the measures (columns) and the time frame for trend estimation (rows). Error bars show 95% confidence intervals. Trends in solid lines are significant at the 0.05 level, while the transparent ones are not, i.e. zero is within the confidence bounds. SD = Standard deviation, quantile‐based measures start with Q, followed by the bounds (e.g. Q95‐05 is the range between the 0.95 and the 0.05 quantile). [file JOC-36-13-s001.pdf]
