# Supplementary material for: Asymmetric trends in seasonal temperature variability in instrumental records from ten stations in Switzerland, Germany and the UK from 1864 to 2012
Source: Int J Climatol. 2015 Apr 2;36(1):13–27. doi: 10.1002/joc.4326 (PMC4950111; doi:10.1002/joc.4326)
Supplement: Supplementary file 2 — Figure S2. Quantile Regression example of slope‐quantile plots. [file JOC-36-13-s002.pdf]

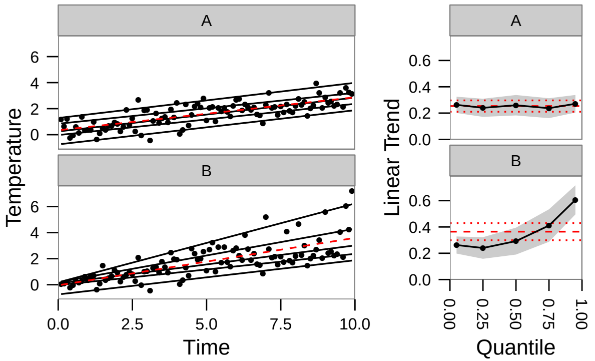

Figure S2: Left: Quantile regression (solid black lines; 0.05, 0.25, 0.5, 0.75 and 0.95 quantile levels) and ordinary least squares regression (OLS, dashed red line) for a set of 99 synthetic temperatures assuming constant variance (top) versus a one-sided increase in variance for higher temperatures (bottom).

Right: Linear trend values for quantile regression estimated at five distinct quantiles 0.05, 0.25, 0.5, 0.75 and 0.95 (connected points with shaded 95% confidence bands) versus trend values for OLS regression (dashed red line with dotted red lines representing 95% confidence bands).
